# Tacrolimus-induced diabetic ketoacidosis with subsequent rapid recovery of endogenous insulin secretion after cessation of tacrolimus: A case report with review of literature

**DOI:** 10.1097/MD.0000000000016992

**Published:** 2019-09-06

**Authors:** Koji Maruyama, Daisuke Chujo

**Affiliations:** a Department of Diabetes, Endocrinology and Metabolism, National Center for Global health and Medicine, Tokyo; b Center for Clinical Research, Toyama University Hospital, Toyama, Japan.

**Keywords:** diabetic ketoacidosis, insulin secretion, interstitial pneumonia, tacrolimus

## Abstract

**Rationale:**

Immunosuppressive agents such as tacrolimus (TAC) and cyclosporin might cause glycemic disorders by suppressing insulin production. However, only a few cases of diabetic ketoacidosis (DKA) with longitudinal evaluation of endogenous insulin secretion related to TAC administration have been reported.

**Patient concerns:**

A 59-year-old Asian woman, who received prednisolone and TAC 4.0 mg for the treatment of anti-aminoacyl-tRNA synthetase antibody-positive interstitial pneumonia, was admitted to our hospital due to impaired consciousness and general malaise.

**Diagnoses:**

She had metabolic acidosis; her plasma glucose, fasting serum C-peptide immunoreactivity (CPR), and urinary CPR levels were 989 mg/dL (54.9 mmol/L), 0.62 ng/mL, and 13.4 μg/d, respectively. No islet-related autoantibodies were detected. Therefore, she was diagnosed with TAC-induced DKA.

**Intervention:**

Intravenous continuous insulin infusion and rapid saline infusion were administered. TAC was discontinued because of its diabetogenic potential.

**Outcomes:**

Sixteen weeks after cessation of TAC administration, she showed good glycemic control without administration of insulin or any oral hypoglycemic agents; her serum CPR level also improved dramatically. These findings suggested that TAC-induced pancreatic beta cell toxicity is reversible.

**Lessons:**

We reported a case of TAC-induced DKA with subsequent recovery of pancreatic beta cell function after cessation of TAC, resulting in good glycemic control. As TAC is widely used, we should pay attention to patients’ glucose levels even though the TAC concentrations used are within the target range. Furthermore, dose reduction or cessation of TAC should be considered if hyperglycemia is detected during administration of this agent.

## 1 Introduction

Tacrolimus (TAC) is widely used as an immunosuppressive agent for suppressing rejection of transplanted organs or tissues and for treating inflammatory diseases, such as nephritic syndrome, rheumatic arthritis, and interstitial pneumonia secondary to polymyositis or dermatomyositis. TAC and cyclosporin have been reported to cause post-transplant diabetes.^[1]^ The diabetogenic potential of TAC is considered to involve suppression of insulin secretion from pancreatic beta cells by inhibiting transcription of the insulin gene, due to the association between TAC and the FK506-binding protein 12 (FKBP-12).^[2]^ Although some cases of TAC-related diabetic ketoacidosis (DKA) have been reported,^[1,3]^ few cases have involved changes in insulin secretion before and after TAC cessation. Herein, we reported a case of DKA induced by TAC, which was being administered for interstitial pneumonia treatment, with subsequent rapid recovery of endogenous insulin secretion after cessation of TAC administration.

## 2 Case report

**Figure 1:**
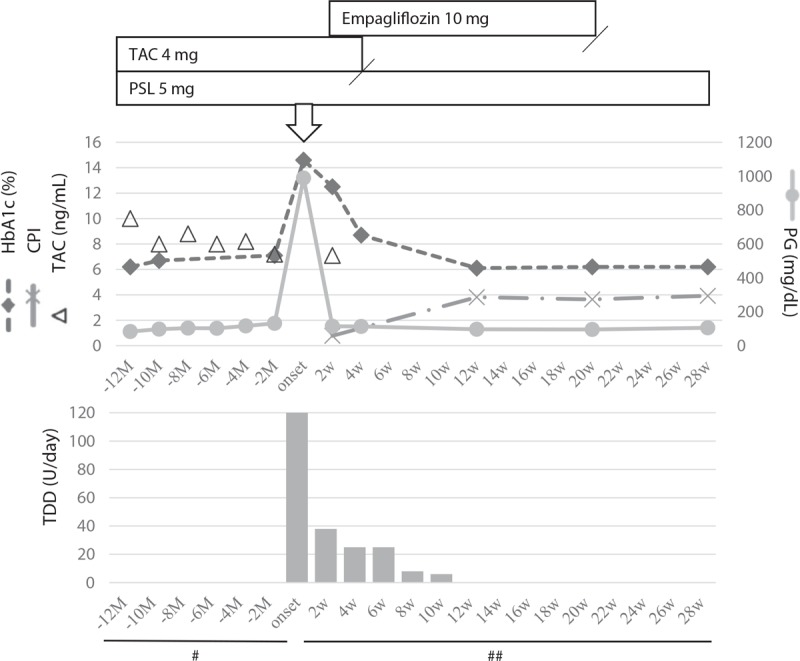
Changes in glycated hemoglobin (HbA1c) levels, C-peptide index, plasma glucose levels, and trough levels of tacrolimus before and after the onset of diabetic ketoacidosis. White arrow indicates the onset of ketoacidosis. ## = each interval is 2 weeks, # = each interval is 2 months, CPI = C-peptide index, HbA1c = glycosylated hemoglobin, PG = plasma glucose, PSL = prednisolone, TAC = tacrolimus, TDD = total daily dose of insulin.

A 59-year-old Asian woman presenting with impaired consciousness and general malaise was brought to our hospital by her family. She had been diagnosed with anti-aminoacyl-tRNA synthetase antibody-positive interstitial pneumonia in February 2017 (13 months before admission) with the complaint of exertional dyspnea. She had never been diagnosed with diabetes; 13 months before admission, her glycosylated hemoglobin (HbA1c) level was 6.1%. On diagnosis of interstitial pneumonia, she started receiving 60 mg of methylprednisolone, followed by 40 mg of prednisolone (PSL) and 4.0 mg of TAC, which was considered effective and well-tolerated therapy for interstitial lung disease with anti-aminoacyl-tRNA synthetase antibody.^[4]^ The dosage of PSL was gradually decreased, and 5.0 mg of PSL and 4.0 mg of TAC had been continued to maintain the remission of interstitial pneumonia. She had no other specific medical history or any significant family history. Following were her vital signs on admission: blood pressure, 110/66 mmHg; respiratory rate, 18 breaths/min; pulse rate, 97 beats/min; and SpO_2_, 96% on room air. Her height was 159 cm and her weight was 90 kg; her body mass index (BMI) was 35.6 kg/m^2^. On physical examination, lungs were clear to auscultation and no significant abnormalities were observed, except slight dehydration of the tongue and axilla. Laboratory data revealed the following: extremely high plasma glucose levels, 989 mg/dL (54.9 mmol/L); presence of urinary ketone bodies; blood pH, 6.85; and bicarbonate level, 2.4 mmol/L; these findings met the diagnostic criteria for DKA proposed by the American Diabetes Association.^[5]^ Fasting serum C-peptide immunoreactivity (CPR) was 0.62 ng/mL and urinary CPR was 13.4 μg/d, indicating deterioration of endogenous insulin secretion. No islet-related autoantibodies were detected (Table 1). Other laboratory data are shown in Table 1. Intravenous continuous insulin infusion and rapid saline infusion were administered; 2 days after admission, insulin administration was switched to multiple daily subcutaneous injections using insulin lispro and insulin glargine 300 mL/unit. The maximum amount of subcutaneous insulin administered was up to 0.9 unit/kg/d. Sixteen days after admission, 10 mg of empagliflozin was also administered for inducing weight reduction. She was discharged with a prescription of 25 units/d of total daily insulin and 10 mg of empagliflozin; the dosage of PSL and TAC were maintained at 5.0 and 4.0 mg, respectively. TAC was discontinued 4 weeks after the onset of DKA as good control of interstitial pneumonia was noted. She did not require insulin lispro and insulin glargine 300 mL/unit to maintain stable glycemic control 4 and 8 days after cessation of TAC, respectively. Finally, although empagliflozin administration was also stopped 16 weeks after TAC cessation, her blood glucose levels were under good control; her HbA1c level was 6.2%, even though PSL administration was continued. Notably, her fasting serum CPR level rapidly recovered to 4.15 ng/mL, which is within the normal range, 8 weeks after TAC cessation and was maintained at similar levels thereafter (Fig. 1). Written informed consent was obtained from the patient for publication of this case report.

**Table 1:**
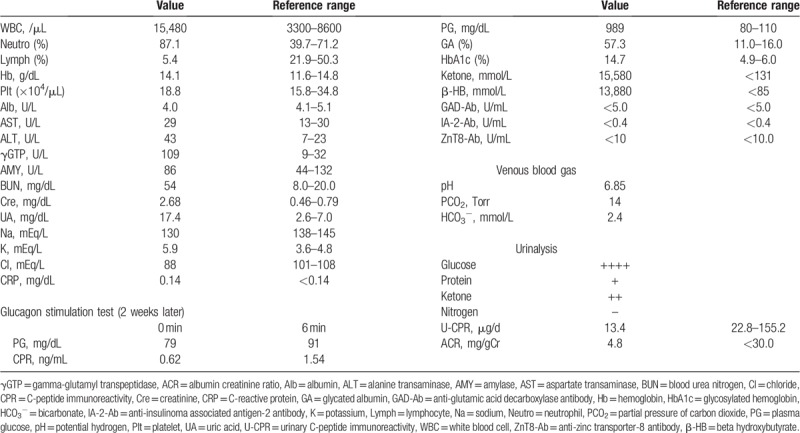
Laboratory data on admission.

## 3 Discussion

TAC or FK506, produced by *Streptomyces tsukubaensis*, was discovered as a macrolide antibiotic in Japan in 1984 and is used as a novel immunosuppressant.^[6]^ FK506 binds to FKBP-12, a cytoplasmic receptor, and this complex inhibits the phosphatase activity of calcineurin, resulting in prohibition of transcription of T-cell activating genes, such as the gene for IL-2.^[7]^ The diabetogenic effect of TAC is predominantly a result of suppression of insulin secretion from pancreatic beta cells and not of deterioration of peripheral insulin sensitivity.^[2,8]^ TAC suppresses transcription of insulin mRNA in beta cells in a time-dependent manner, leading to decreased insulin production; however, it does not affect pancreatic alfa cells.^[2]^ In addition, TAC has also been reported to cause decreased expression of the insulin promoter chloramphenicol acetyltransferase (CAT) reporter gene.^[9]^

Due to this diabetogenic potential of TAC, 7.1% of patients with interstitial pneumonia secondary to polymyositis and dermatomyositis receiving TAC developed newly-diagnosed diabetes during a 3-year investigation period in Japan.^[10]^ Although it is unclear why only a few TAC-receiving individuals develop DKA, 15 cases of TAC-induced DKA have been reported after transplantation.^[1]^ Mean age of these patients was 29.9 years; 47% of them were men; average BMI was in the normal range (22.1 ± 4.7 kg/m^2^) and 40% developed DKA within the first 3 months of transplantation. Table 2 shows details of the TAC-induced DKA cases that involved diseases other than transplantation. Out of 4 patients (including our patient), 3 had kidney diseases, such as focal segmental glomerulosclerosis,^[3]^ minimal change glomerulopathy,^[11]^ and lupus nephritis.^[12]^ To the best of our knowledge, this is the first case of TAC-induced DKA in which TAC was used for treating interstitial pneumonia. These 4 patients developed DKA despite receiving <10 ng/mL of TAC, indicating appropriate trough levels for minimizing diabetes risk.^[13]^ TAC effect on insulin secretion is considered dose- and time-dependent in vitro.^[9]^ The maximal decrease in insulin secretion is seen after 72 hours, and no further decrease occurred.^[9]^ According to that data, we assumed that insulin secretion ability was suppressed several days after TAC administration. However, we could not find the constant tendency regarding dose or time before the onset of DKA among the cases including ours. Our case was also administrated PSL and gained approximately 10 kg weight gain since PSL administration had been initiated, leading to insulin resistance increase. It may depend on the balance between suppressed insulin secretion by diabetogenic drugs and insulin resistance caused by obesity or other factors when DKA occurred. Although high BMI combined with TAC and PSL administration might contribute to the development of DKA, it is difficult to speculate further mechanisms due to the lack of such cases. However, the expression levels of calcineurin and FKBP-12 in pancreatic endocrine cells should be considered. Because pancreatic alfa cells contain more calcineurin and fewer FKBP-12 than beta cells, glucagon secretion from alfa cells is not affected by TAC.^[2]^ Differences in calcineurin and FKBP-12 expression levels among individuals might affect the amount of insulin secretion, leading to differences in diabetogenic effects of TAC.

**Table 2:**
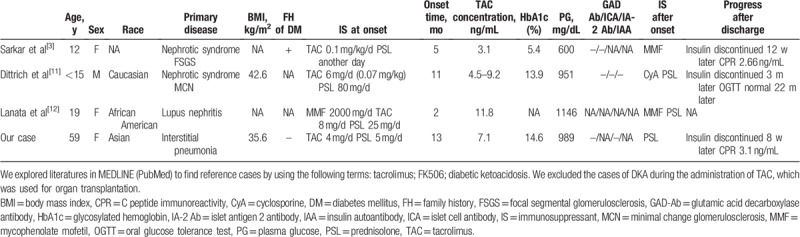
List of cases of tacrolimus-induced diabetic ketoacidosis.

If diabetes occurs during TAC therapy, physicians should consider dose reduction, cessation of TAC administration, and use of alternative agents, such as cyclosporine, that have less diabetogenic potential than TAC.^[14]^ Additionally, a rapid tapering or cessation of corticosteroid also should be considered.^[15]^ The diabetogenic effect of TAC is usually considered to be dose dependent and reversible.^[2]^ Both insulin production and mRNA transcription completely recovered 7 days after the cessation of TAC in vitro; insulin secretion recovered when TAC concentration became <0.09 ± 0.11 ng/mL in rats.^[2]^ Furthermore, Sarkar et al^[3]^ reported a patient who showed recovered insulin secretion 12 weeks after cessation of TAC administration. In our case, endogenous insulin secretion and glycemic control improved dramatically 8 weeks after TAC withdrawal, consistent with previous reports.^[3,15]^ Thus, our case also demonstrated that TAC-induced pancreatic beta cell toxicity is reversible and that insulin secretion recovers within several weeks of discontinuing TAC in vivo.

Since TAC is not only used as an immunosuppressant after organ or tissue transplantation but also for treating various inflammatory diseases, blood sugar levels should be routinely monitored in patients on TAC based on this case report and reported literatures. TAC-induced glycemic disorders including DKA might be life-threatening, even when trough levels of the drug are within the target range. In the future, accumulation of similar cases is necessary to analyze associated risks and background characteristics of TAC-induced DKA.

## 4 Conclusion

We reported a case of DKA induced by TAC, used for treating interstitial pneumonia, involving subsequent dramatic recovery of insulin secretion after cessation of TAC. As TAC is widely used for treating various diseases, attention should be paid to glycemic disorders, even though TAC concentrations are within the target range.

## Acknowledgments

This study was not funded by any grants. The authors would like to thank Editage (Tokyo, Japan; www.editage.jp) for English language editing.

## Author contributions

**Supervision:** Daisuke Chujo.

**Writing – original draft:** Koji Maruyama.

**Writing – review & editing:** Koji Maruyama, Daisuke Chujo.
